# Supplementary material for: Transcriptome Analysis Reveals Vimentin-Induced Disruption of Cell–Cell Associations Augments Breast Cancer Cell Migration
Source: Cells. 2022 Dec 13;11(24):4035. doi: 10.3390/cells11244035 (PMC9776984; doi:10.3390/cells11244035)
Supplement: Supplementary file 1 [file cells-11-04035-s001.zip › DEGs and enrichment analysis.zip!/DEGs and enrichment analysis/WT_VIMvsVector KEGG/UP/WT_VIMvsVector_up.dot.pdf]

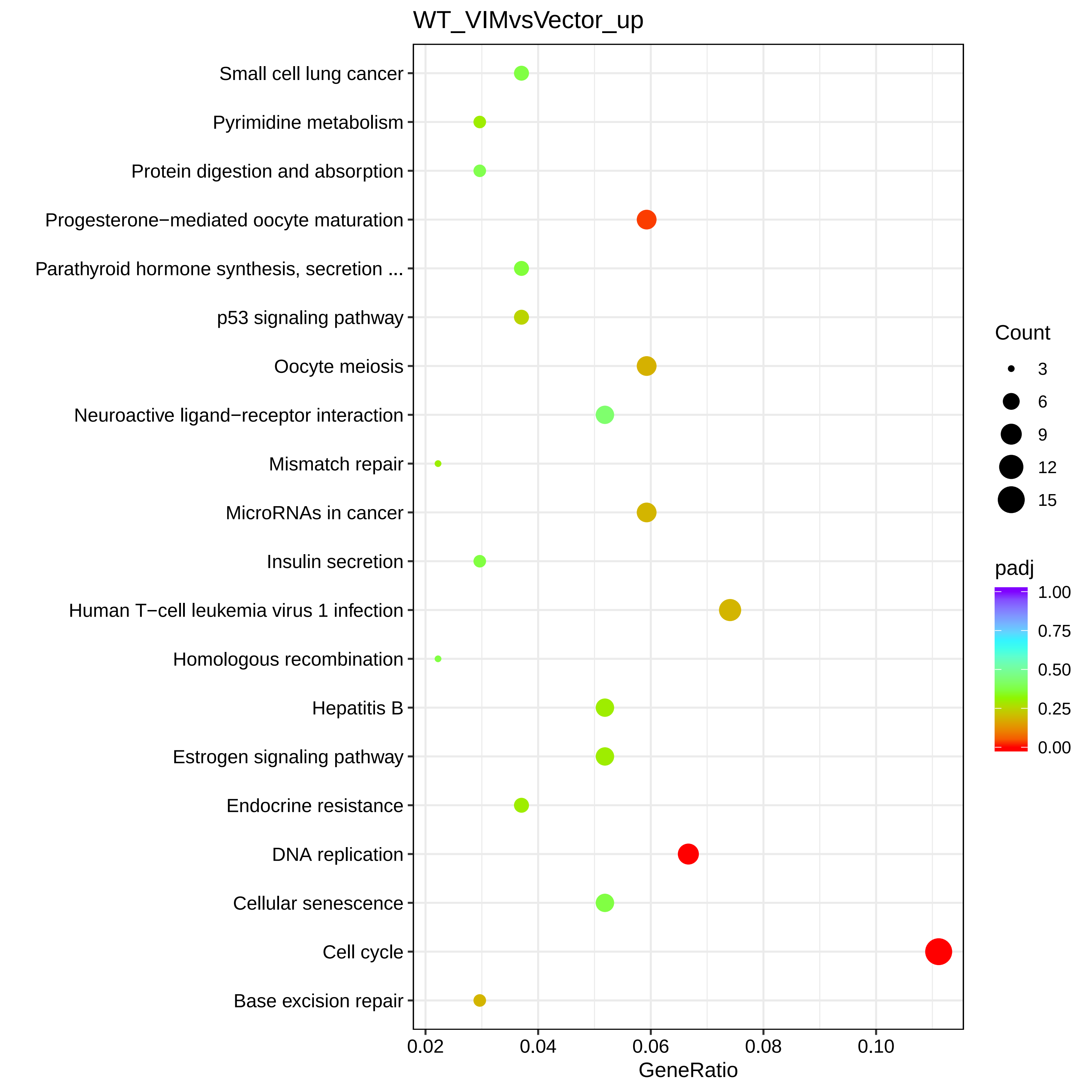

WT_VIMvsVector_up
Small cell lung cancer
Pyrimidine metabolism
Protein digestion and absorption
Progesterone−mediated oocyte maturation
Parathyroid hormone synthesis, secretion ...
p53 signaling pathway
Oocyte meiosis
Neuroactive ligand−receptor interaction
Mismatch repair
MicroRNAs in cancer
Insulin secretion
Human T−cell leukemia virus 1 infection
Homologous recombination
Hepatitis B
Estrogen signaling pathway
Endocrine resistance
DNA replication
Cellular senescence
Cell cycle
Base excision repair
0.02
0.04
0.06
0.08
0.10
GeneRatio
Count
3
6
9
12
15
padj
1.00
0.75
0.50
0.25
0.00
